# Trypanosomatid histones: the building blocks of the epigenetic code of highly divergent eukaryotes

**DOI:** 10.1042/BCJ20240543

**Published:** 2025-03-14

**Authors:** Josefina Ocampo, Santiago Carena, María del Rosario López, Valentina Sol Vela, Romina Trinidad Zambrano Siri, Sofia Antonella Balestra, Guillermo Daniel Alonso

**Affiliations:** 1 Instituto de Investigaciones en Ingeniería Genética y Biología Molecular “Dr. Héctor N. Torres” (INGEBI), Consejo Nacional de Investigaciones Científicas y Técnicas (CONICET), Buenos Aires, Argentina; 2 Departamento de Fisiología, Biología Molecular y Celular, Facultad de Ciencias Exactas y Naturales, Universidad de Buenos Aires, Buenos Aires, Argentina

**Keywords:** canonical histones, histone code, histone PTMs, histone variants, trypanosomatids

## Abstract

Histones play a fundamental role in eukaryotic organisms not only as scaffolding proteins in DNA packaging but also in regulating gene expression. They constitute the protein reel around which DNA wraps forming nucleosomes. This initial packing gives rise to the chromatin fiber which is next folded into three-dimensional arrangements. Additionally, histones have expanded their functions through the emergence of histone variants which have specialized purposes and can deeply affect chromatin organization and dynamics. Moreover, both canonical histones and histone variants comprise the building blocks of the histone code by being targets of different post-translational modifications (PTMs) that occur in a highly regulated manner both in place and time. Most of the above-mentioned about chromatin organization is conserved among eukaryotes. However, trypanosomatid histones have many peculiarities that entail a special description. In this review, we compile the current knowledge of canonical core histones, histone variants, and their PTMs in trypanosomatids. We highlight the similarities and differences between histone variants and their canonical counterparts in trypanosomatids, and we compare them with those from model organisms. Finally, we discuss the crosstalk between different histone marks and their genomic distribution underlying the uniqueness of trypanosomatids.

## Introduction

Histones are proteins involved in DNA wrapping to form nucleosomes, in essence in all eukaryotes. There are four highly conserved canonical histones (H2A, H2B, H3, and H4), whose synthesis is coupled to DNA replication. Histones have traditionally been considered highly conserved proteins. However, throughout evolution, histone variants have evolved serving a variety of specialized functions by replacing their canonical counterparts at specific regions of the genome. Therefore, histones have diversified their roles in key biological processes such as chromosome segregation, DNA replication, transcriptional regulation, and DNA repair. Among the basis of this expansion of functionalities, they present changes in sequence composition, post-translational modifications (PTMs), and the acquisition of specialized domains. These variations provide additional properties to diversify protein–protein and protein–DNA interactions. While canonical histones are encoded by multicopy genes and organized in clusters, histone variants are encoded by single genes. Another difference between them is that while canonical histones are only synthesized during the S-phase of the cell cycle and do not contain introns, histone variants are constitutively expressed and incorporated into chromatin independently of DNA replication displaying specialized functions [1].

H4 is the histone with the greatest evolutionary constraint and, for a long time, was considered an invariant histone [2]. However, in 1992, an H4 variant was described in *Trypanosoma brucei* [3]. More recently, a novel H4 variant was detected in Hominidae whose expression was tumor-stage-dependent in breast cancer [4]. Although H2B is less evolutionarily constrained than H4, it has a limited specialization given by the reduced number of variants, some of them occurring in mammals, Apicomplexa parasites [5], and Trypanosomes [6]. On the other hand, H2A and H3 have evolved to attend diversified functions, getting involved in epigenetic silencing, gene expression, and centromere function, giving rise to an extensive number of variants [2].

Apart from the existence of histone variants, histones are frequently chemically modified by PTMs that can occur in the carboxy (C) or amino (N) terminal tails or in the globular domain. Although any known PTM can occur in histones, the most frequent ones include acetylation, methylation, phosphorylation, ubiquitylation, and ADP-ribosylation [7]. These modifications have an important impact on transcription, replication, DNA repair, and chromosome condensation [8,9].

Trypanosomatids are a family of unicellular protozoan parasites that cause millions of infections all over the world affecting human and other mammal hosts. *Trypanosoma cruzi*, *T. brucei,* and *Leishmania major* belong to this group and are commonly denominated TriTryps. They are causal agents of Chagas disease, sleeping sickness, and leishmaniasis, respectively.

In TriTryps, chromatin has a similar global composition to other eukaryotes but presents some peculiarities that entail a specialized approach. They count on the four canonical histones mentioned above, the *linker* histone H1 and histone variants [10]. Within their histone variants: H2A.Z, H2B.V, and H3.V are conserved among TriTryps, but H4.V is only present in *T. brucei* [6]. Histone H1 is unique because it lacks a globular domain [11]. This peculiarity might be of great importance for global chromatin folding. However, the discussion of H1 demands a specialized issue. Here, we will focus on the core histones and their variants.

In general, histones are highly conserved proteins and histone variants are less conserved than canonical ones [12]. In model organisms, they are responsible for the regulation of gene expression, since histone variants and histone PTMs constitute the founding elements of the histone code [13].

One peculiarity of trypanosomatids is that their genes are organized in long transcriptional units and are constitutively transcribed into polycistrons that later mature into monocistronic units by a process known as trans-splicing. Therefore, trypanosomatid histone synthesis is fundamentally different from that in other eukaryotes since the levels of histone mRNAs increase during the S phase of the cell cycle despite the transcription rate of their polycistronic transcriptional units (PTUs) remains unchanged, probably balanced by post-transcriptional regulation [14–16].

Additionally, both canonical and variant histones are dissimilar in charge, sequence and PTMs compared with those of model organisms [10,17,18]. Moreover, in *T. brucei,* it was recently shown that H2A and H2B are the most divergent histones and these differences impact nucleosome structure [19].

Given that the regulation of gene expression in trypanosomatids occurs mainly post-transcriptionally, for a long time, histones and their interacting proteins were poorly explored in TriTryps. Nevertheless, over the last decade several studies using proteomic approaches have shed light into this field by exploring histone PTMs throughout the life cycle of the parasite in *T. cruzi* [20–23] and *T. brucei* [24,25]. In parallel, several genomic studies, aiming to expose the epigenetic code, have described the location of the histone marks in their genomes and tried to decipher their combinatorial roles [6,26–30]. Furthermore, some functional studies were performed attempting to unveil the role of chromatin punctuation and their implications [31–34]. In this review, we make a detailed description of the canonical histones and histone variants present in TriTryps. Then, we discuss histones PTMs detected by proteomic studies and other techniques. Finally, we bring together proteomic studies, genomic analysis, and the functional information of histones available for each TriTryp, highlighting their roles and cross-talk among different epigenetic marks.

For simplicity, we will use the prefixes Tc, Tb, or Lm for *T. cruzi*, *T. brucei,* and *L. major,* respectively.

## Histone variants and their canonical counterparts in TriTryps

### A well-conserved histone H4 and the most variant histone H2B.V

While H2A and H2A.Z are common to every eukaryote, trypanosomatid H2B.V, H3.V appeared later in evolution and H4.V emerged only in *T. brucei* [6,10,35].

Trypanosomatid histones are, in general, well conserved among TriTryps, H4 being the most conserved and H2A/H2B the most variable ones, especially H2B [19]. As expected, the histones of *T. cruzi* and *T. brucei* share a greater degree of conservation, while those from *L. major* are the least conserved [10] and their main differences reside in the N-terminal tails [36].

**Figure 1:**
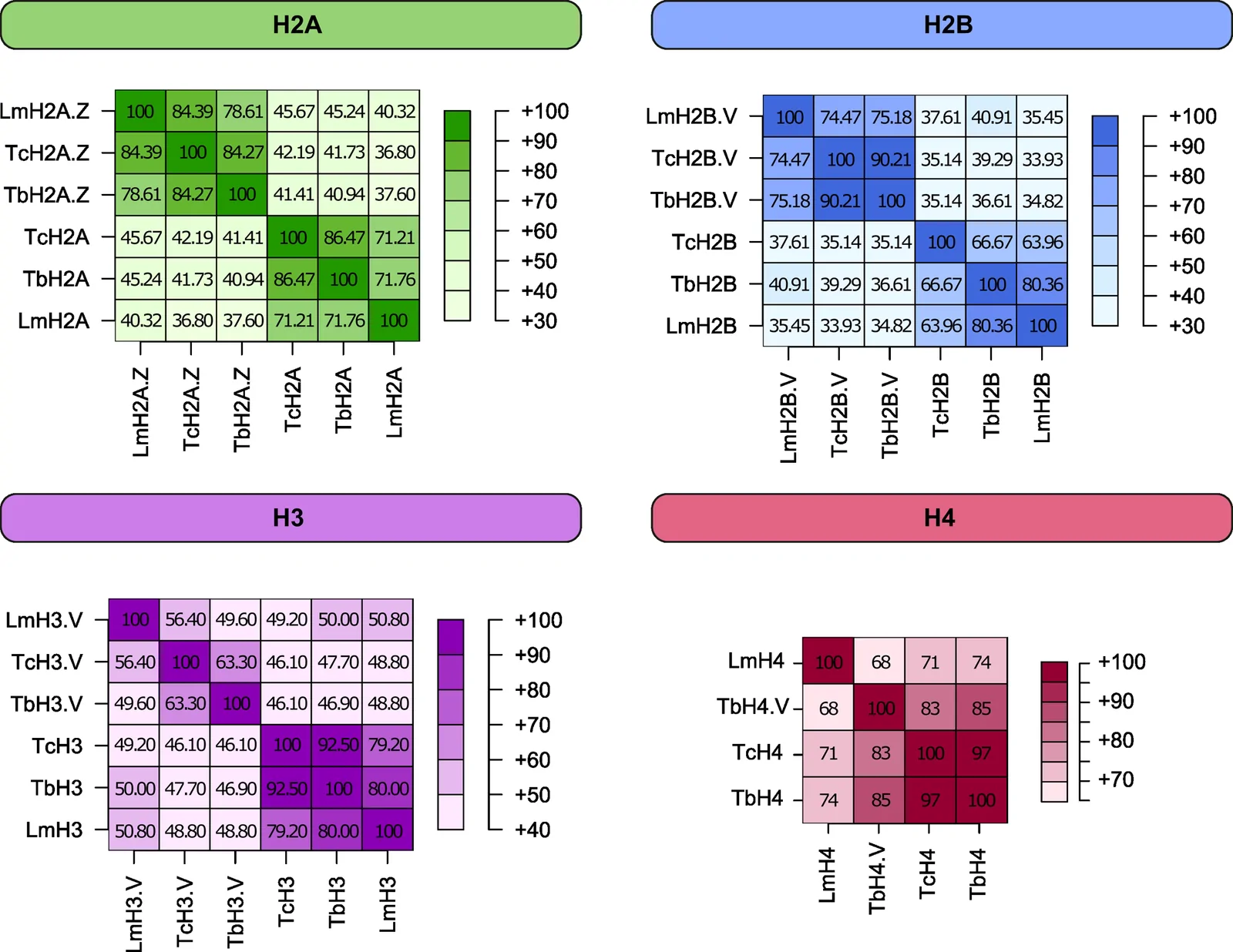
Identity matrix for canonical and variant histones in TriTryps. Identity matrixes were generated from multiple sequenced alignments (MSA) using Clustal Omega (https://www.ebi.ac.uk/jdispatcher/msa/clustalo) and plotted with R. The following sequences were used: *L. major* Friedlin: H2A (LmjF.21.0915) and H2A.Z (LmjF.17.0280); *T. cruzi* Dm28c: H2A (BCY84_17384) and H2A.Z (C4B63_61g127)*; T. brucei* brucei TREU927: H2A (Tb927.7.2820) and H2A.Z (Tb927.7.6360); *L. major* Friedlin: H2B (LmjF.28.0210) and H2B.V (LmjF.28.0210); *T. cruzi* Dm28c H2B (BCY84_06298) and TcY H2B.V (TcYC6_0044700); *T. brucei* brucei TREU927: H2B (Tb927.11.7350); *L. major* Friedlin: H3 (LmjF.10.0870) and H3.V (LmjF.19.0630); *T. cruzi* Dm28c H3 (BCY84_02623) and H3.V (BCY84_18558); *T. brucei* brucei TREU927: H3 (BCY84_02623) and H3.V (Tb927.10.15350); *L. major* Friedlin: H4 (LmjF.06.0010); *T. cruzi* Dm28c H4 (BCY84_15616); *T. brucei* brucei TREU927: H4 (Tb927.5.4260) and H4.V (Tb927.2.2670).

Additionally, unlike many model organisms that do not have a specialized H2B, TriTryps possess a histone variant, H2B.V. Remarkably, the sequence conservation of H2B.V among TriTryps is higher than that observed for H2B. On the other hand, H2A and H3 have diversified their functions with the emergence of H2A.Z and H3.V that display specialized functions at regions where transcription initiates or terminates, respectively [6,29], expanded below. It is worth noting that LmH2A has a similar percentage of identity either to TcH2A or to TbH2A, but LmH2A.Z resembles TcH2A.Z more than TbH2A.Z. Additionally, the H3.V variant is less conserved among TriTryps than canonical H3 (Figure 1).

Comparing variant histones with their canonical counterparts, TbH4.V/TbH4 shares the highest percentage of sequence identity (85%) and, when a substitution occurs, the chemical nature of the amino acid residue is preserved. Comparing H2A/H2A.Z and H3/H3.V shows that their identities are close to 50% in every case, with their main differences located in the N-terminal region. The biggest variations are observed when comparing the H2B.V/H2B pair (ranging from 33% to 39% in each TriTryp). The most notable feature is that H2B.V has a longer C-terminal tail than canonical H2B with potential implications for their roles, as discussed below. The differences observed between histone variants and their canonical counterparts suggest a way to modulate nucleosome structure with potential implications for higher-order folding and DNA accessibility.

## PTMs and the most remarkable features of each histone kind in TriTryps

### An extended N-terminal tail in H2A.Z is only conserved in TriTryps

**Figure 2:**
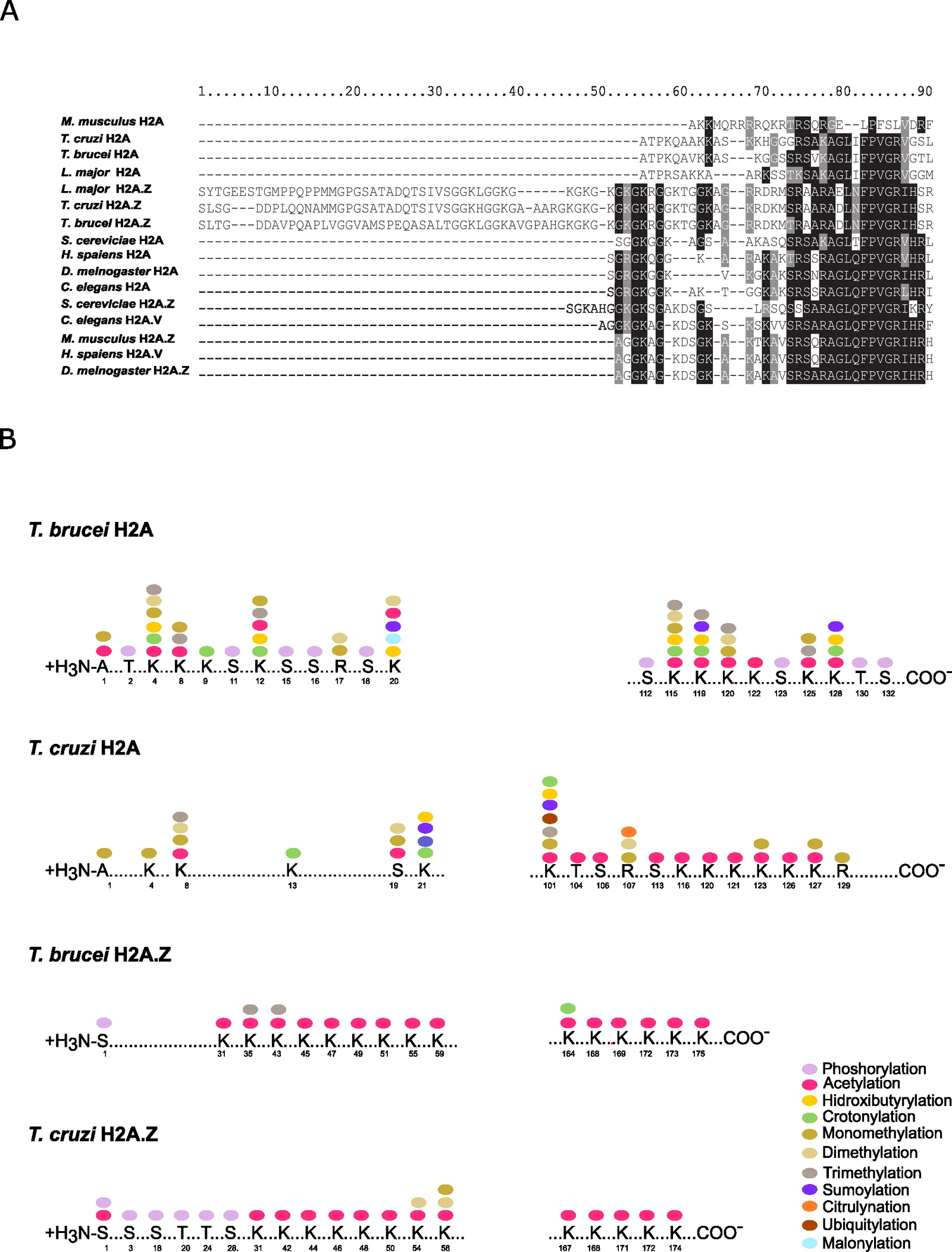
Extended H2A.Z N-terminal tail and PTMs. (**A**) Alignments and shadings were performed with ClustalW and Boxshade 3.3, respectively. The following sequences were used: *L. major* Friedlin: H2A (LmjF.21.0915) and H2A.Z (LmjF.17.0280); *T. cruzi* Dm28c: H2A (BCY84_17384) and H2A.Z (C4B63_61g127)*; T. brucei* brucei TREU927: H2A (Tb927.7.2820) and H2A.Z (Tb927.7.6360); *S. cerevisiae* H2A.1 (1 NP_010511.3) and H2A.Z (Z-NP_014631.1); *Drosophila melanogaster:* H2A (NP_724343.1) and H2A.Z (NP_524519.1); *C. elegans*: H2A (NP_010511.3) and H2A.V (NP_500569.1); *H. sapiens*-H2A-type1 (CCDS4619.1) and *H. sapiens* H2A.V (CCDS47581.1); *M. musculus:*H2A-like-1 (CCDS53003.1) and H2A.Z (CCDS38647.1). Only 90 aminoacidic residues are shown. (**B**). Schematic overview of the PTMs detected at the N- and C-terminal tails in H2A and H2A.Z of *T. cruzi* and *T. brucei*. The distance between residues is not represented on a real scale. Different colors indicate different PTMs as detailed in the chart.

In TriTryps, the only H2A variant is H2A.Z which was first identified in *T. brucei* simultaneously with H2B.V as partners for dimerization [37]. When comparing H2A with H2A.Z in each TriTryp, the degree of conservation is similar, ranging from 40% to 42% of identity, with the main differences given by the presence of a longer N-terminal tail in H2A.Z in every case (Figures 1 and 2A). This sequence extension harbors numerous amino acid residues that could be targets of PTMs. This is also the most notable difference when comparing H2A and H2A.Z from TriTryps with those from model organisms. The precise role of this extension is poorly explored, but its main impact is probably led by the presence of acetylatable residues.

In *T. cruzi* and *T. brucei*, both H2A and H2A.Z possess several amino acid residues that could be post-translationally modified, specially enriched at their N- and C-terminal tails, but in canonical H2A, some PTMs were also detected within the globular domain. In particular, these histones are highly acetylated on the C-terminal tail. In the case of H2A.Z, the presence of acetylatable and phosphorylatable residues in the N-terminal tail is also noteworthy. Here, we compiled the PTMs detected in *T. cruzi* during metacyclogenesis and in epimastigotes, and for *T. brucei* procyclic and blood forms from different proteomic studies [22–25] (Figure 2B). The role of only a few of these PTMs has been studied so far, and we will discuss their potential implications below.

Some PTMs found in H2A.Z seem to be unique in TriTryps. Such is the case for H2A.ZK54ac and H2A.ZK58ac. Although those lysine residues are conserved among eukaryotes, their acetylation has only been detected in trypanosomes [21,24,25]. Histone acetylations are normally involved in facilitating transcription activation [38]. These TriTryp-specific acetylations might be involved in some trypanosome-specific pathway or might help to potentiate the almost constitutive actively transcribed chromatin state characteristic of these organisms.

Some eukaryotes depend on histone variant H2A.X, which is phosphorylated in response to DNA damage (γH2Ax) [39]. Trypanosomes do not have an H2A.X variant, but in *T. brucei,* phosphorylation of canonical H2A at threonine 130 was described to have a homologous role [40].

### The H2B.V of TriTryps resembles H2B from model organisms more closely than its canonical counterpart

Histone H2B associates with histone H2A to form heterodimers. Additionally, four-helix bundle motives are established between H2B and H4 to connect with the H3–H4 tetramer to form the nucleosome.

Most eukaryotes usually encode several isoforms of H2B in their genomes, but H2B specialization includes only a few variants [2]. Among H2B variants, apicomplexan and trypanosomatids possess H2B.Z and H2B.V, respectively [5,37,41].

When comparing canonical H2B from TriTryps with those of model organisms, the most remarkable difference is the shorter N-terminal tail in trypanosomes and *Leishmania*. Unlike H2B, TriTryps H2B.V has an extended N-terminal tail and the sequence composition, or the chemical nature of the amino acid residues is preserved to some extent compared with other eukaryotes. This observation suggests that some of the missing residues in TriTryps H2B might have a homologous function in TriTryps H2B.V. Such is the case for *S. cerevisiae* ScH2BK123 whose equivalent residue is TbH2B.VK129 [42]. Additionally, the most notable feature is that H2B.V has a longer C-terminal tail than canonical H2B in every TriTryp, and this extension is essential for cell viability [42].

**Figure 3:**
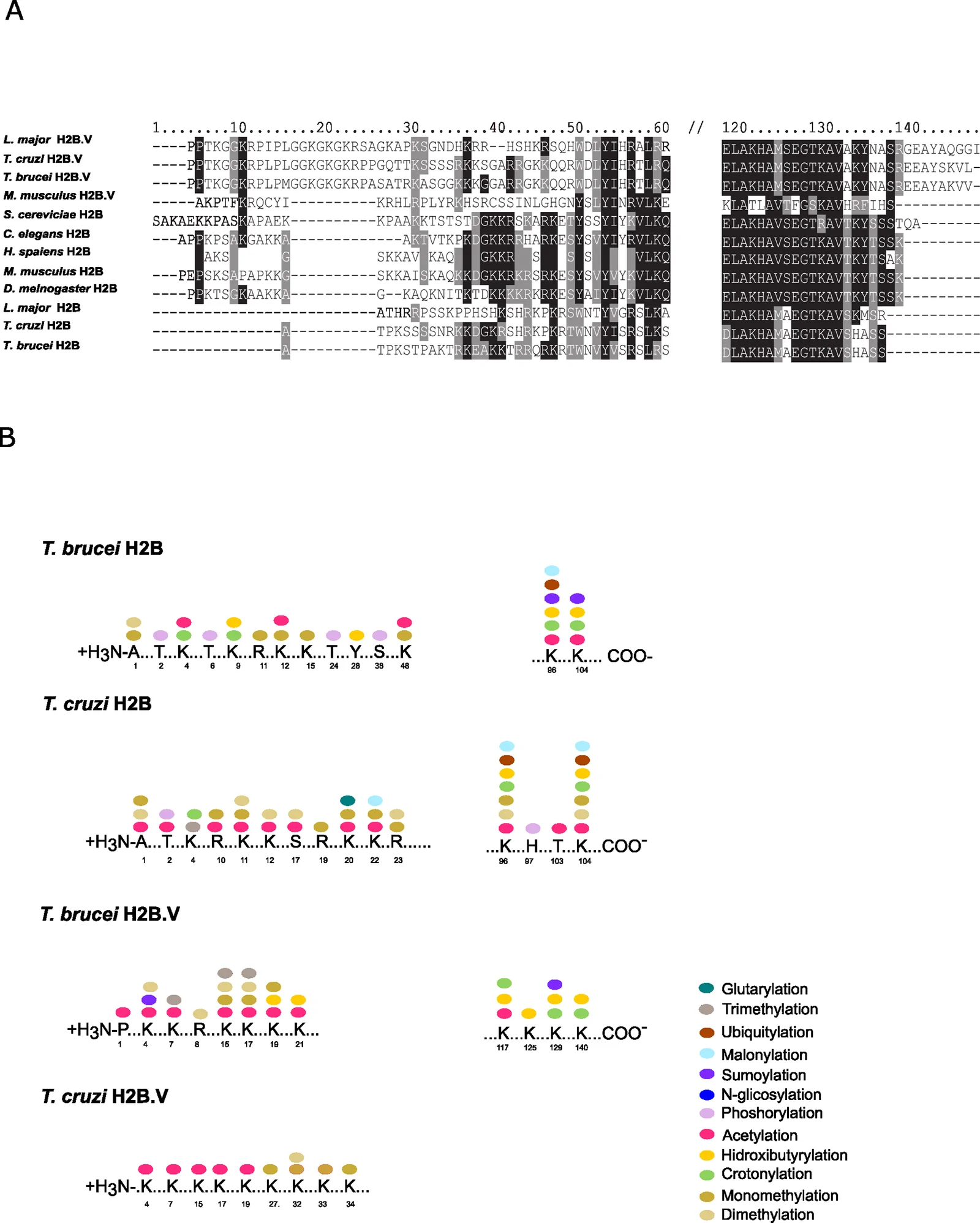
Conserved H2B.V among TriTryps with diversified PTMs. (**A**) Alignments and shadings were performed with ClustalW and Boxshade 3.3, respectively. The following sequences were used: *L. major* Friedlin: H2B (LmjF.28.0210) and H2B.V (LmjF.28.0210); *T. cruzi* Dm28c H2B (BCY84_06298) and TcY H2B.V (TcYC6_0044700); *T. brucei* brucei TREU927: H2B (Tb927.11.7350) and H2B.V (Tb927.11.7350); *S. cerevisiae:* H2B.1 (NP_010510.3); *Drosophila* H2B (NP_724342.1); *C. elegans*-H2B (NP_505464); H. *sapiens* H2B (NP_066402.2); *M. musculus* H2B (NP_783595.1) and H2B.V (CCDS49342.1). Only the N- and C-terminal tails are shown with the numbering based on *S. cervisiae* sequence. (**B**). Schematic representation of the PTMs detected at the N- and C-terminal tail in *T. cruzi* and *T. brucei* H2B and H2B.V. The distance between residues is not represented on a real scale. Different colors indicate different PTMs as detailed in the chart.

TriTryps H2B.V also have low sequence conservation when compared to H2B variants from model organisms. As is true of the other histones, most of the differences in their primary sequences reside in the N-terminal tail (Figure 3A).

H2B and H2B.V not only differ in their primary sequences but they are also targets of different PTMs. The most updated proteomic maps described more than 35 modified residues in TcH2B and 17 for TcH2B.V. While TcH2B tends to be hypermethylated, hyperacetylations prevail in TcH2B.V [22]. In the case of TcH2B, PTMs are spread throughout the entire protein, while in TcH2B.V, they are mainly located in the N-terminal tail (Figure 3B).

TbH2B differs from TcH2B in that it has different combinations of PTMs. The peculiarities observed in TriTryps H2B include mono-methylation of the N-terminal alanine, which seems to be an exclusive modification of TriTryps. Even though it was first described in *T. brucei,* it was also detected in *T. cruzi* later on [22,43]. Some H2B PTMs are common to all trypanosomatids, and some seem to be strain-specific. The common PTMs in *T. cruzi* and *T. brucei* include H2BA1me, H2BA1me2, and H2BK12ac. On the other hand, H2BK4 acetylation was only detected in *T. brucei,* and H2BS63 phosphorylation was only detected in *T. cruzi* [21,44]. Nevertheless, considering that K in position 4 is a conserved residue in *T. cruzi*, there is a chance that in different experimental conditions, this acetylation might be detected. Besides, in some cases, the modified residue is conserved among TriTryps but is a target of different modifications in each parasite. That is the case for H2BK4: while being acetylated in *T. brucei*, it is trimethylated in *T. cruzi* epimastigotes [21,22]. That is also the case for the highly modified residues H2BK96 and H2BK104; they are the target of some common PTMs and some others that seem to be strain-specific [22,24,45]. The roles of H2B PTMs still remain unknown, but some of them are probably involved in life stage-specific modulations, considering they were only detected in specific life forms of the parasites. Among these stage-specific marks, H2BK4me3 and H2BK11me1 were only detected in epimastigotes [21]. The role of H2B ubiquitylation at the C-terminal tails in TriTryps remains still unknown, but in yeast, it is involved in silencing [46].

On the other hand, both TbH2B.V and TcH2B.V have a hyperacetylated N-terminal tail [20,22,24]. Enticingly, despite the conservation in the number of modified residues in H2B.V among Trypanosomes, PTMs seem to be extensively diversified between strains. While in *T. cruzi,* they are clustered in the N-terminal tail, in *T. brucei* they are spread all along the protein [22,24] (Figure 3B). The role of H2B.V acetylation is still unexplored in TriTryps. In *T. gondii,* the acetylated N-terminal tail of H2B.Z is important for interactions with specific protein partners related to chromosome maintenance/segregation and cell cycle progression [47].

### H3K76 differential methylation controls cell cycle progression in *T. cruzi* and *T. brucei*

Histone H3 could be called a scaffolding histone. Not only does it form a homodimer with the second H3 molecule but it also interacts with H4, with H2B, and with the DNA. Furthermore, during *in vitro* nucleosome reconstitution, the [H3–H4]_2_ tetramers are the first step of nucleosome assembly [48,49].

**Figure 4:**
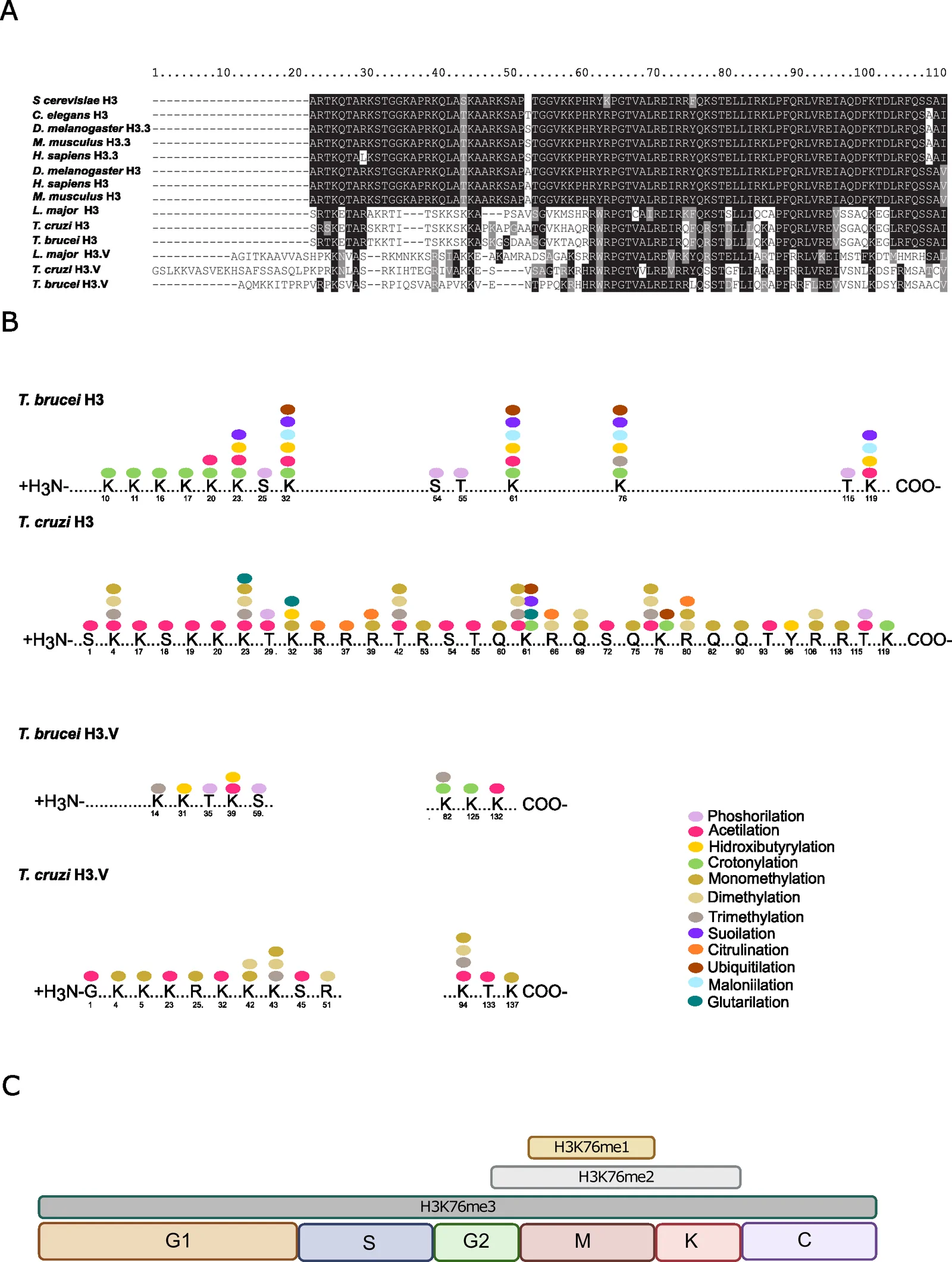
Acetylation and differential methylation states are the dominant PTMs on histone H3. (**A**) Alignments and shadings were performed with ClustalW and Boxshade 3.3, respectively. The following sequences were used: *L. major* Friedlin: H3 (LmjF.10.0870) and H3.V (LmjF.19.0630); *T. cruzi* Dm28c H3 (BCY84_02623) and H3.V (BCY84_18558); *T. brucei* brucei TREU927: H3 (Tb927.1.2430) and H3.V (Tb927.10.15350) *S. cerevisiae:* H3 (NP_009564.1); Drosophila H3 (NP_001027285.1) and H3.3 (NP_511095.1); *C. elegans*-H3 (NP_509344.1); H*. sapiens* H3 (NP_003520.1) and H3.3 (AAH81561.1); *M. musculus* H3 (NP_659539.1) and H3.3 (NP_032236.1). SSchematic representation of (**B**) Thethe distribution of the most prevalent PTMs detected on H3 and H3.V. The distance between residues is not represented on a real scale. Different colors indicate different PTMs as detailed in the chart.; and (**C**) the differential methylation states of H3K76 detected along the cell cycle in TriTryps.

Although H3, together with H4, is a slow-evolving histone, several variants of H3 have emerged to diversify its functions [2]. In general, model organisms have more than one kind of H3 variant, and most eukaryotes possess a specialized centromeric histone. TriTryps present only one H3 variant, H3.V [50], that is not a conspicuous homolog of any variant present in other eukaryotes since they differentiated from each other independently early during evolution [10]. Another consequence of this early divergence of trypanosomatid histones is that H3 lacks the ‘KAPRK’ motive usually present in the globular domain in model organisms and has many amino acid changes in the N-terminal tail (Figure 4A). Consequently, experimental immunodetection of trypanosomatid H3 requires the production of customized antibodies.

Adding to the complexity, H3 and H3.V are targets of different PTMs, some of them are conserved among eukaryotes, some are TriTryp-specific, and some seem to be strain-specific. In *T. cruzi* and *T. brucei,* H3 is more extensively modified compared to H3.V [22,24]. Both H3 and H3.V are mainly acetylated in the N-terminal domains, while methylations predominate in the globular domain [51]. In TcH3*,* 32 modified residues were detected distributed all along the protein, while in TcH3.V, only 13 modified residues were detected, mainly located in the N-terminal tail [22,23]. In TbH3, 26 modified residues were detected and in TbH3.V, only 11 evenly spread in the entire protein in both cases [24,25]. The complexity is further expanded given that each modified residue might be the target of several PTMs (Figure 4B).

Three modified residues conserved in H3 among TriTryps include lysines 4, 23, and 76. Acetylation of these residues (H3K4ac, H3K23ac, and H3K76ac) has been detected [22,44,52]. H3K4me3 was detected in proteomic studies and was mapped at putative transcription start regions in *T. brucei, T. cruzi,* and *L. tarentolae* [29,30,42]. H3K4 and H3K23 can also be di-methylated. Specially, TcH3K23me2 was only detected in nonproliferative stages [21].

A central role is taken by H3K76 methylation, homologous to H3K79 in model organisms, which can be mono, di, or trimethylated (H3K76me, H3K76me2, and H3K76me3) by DOT1 enzymes. In other eukaryotes, this residue is modified by a unique DOT1 isoform, but in trypanosomatids, two isoforms perform this work. In both parasites, H3K76 differential methylation controls cell cycle progression with some peculiarities in each case. A common feature for *T. cruzi* and *T. brucei* is that trimethylation occurs throughout the cell cycle [21,32,33,53] (Figure 4B). Moreover, a conserved cell cycle-regulated pattern was recently shown for the equivalent residue H3K73 in *Leishmania mexicana* [54].

### H4.V could have arisen to fulfill the physiological uniqueness of *T. brucei*

H4 is the most conserved histone among eukaryotes, especially its C-terminal tail, and coevolved with histone H3 to form [H3–H4]_2_ tetramers. Like H2B, H4 histones do not interact as homodimers in the nucleosome and have very few variants in every organism. This high conservation and slow evolution of histone H4 may be necessary to fulfil its role, making contact with the other three histones to form the nucleosome [2].

**Figure 5:**
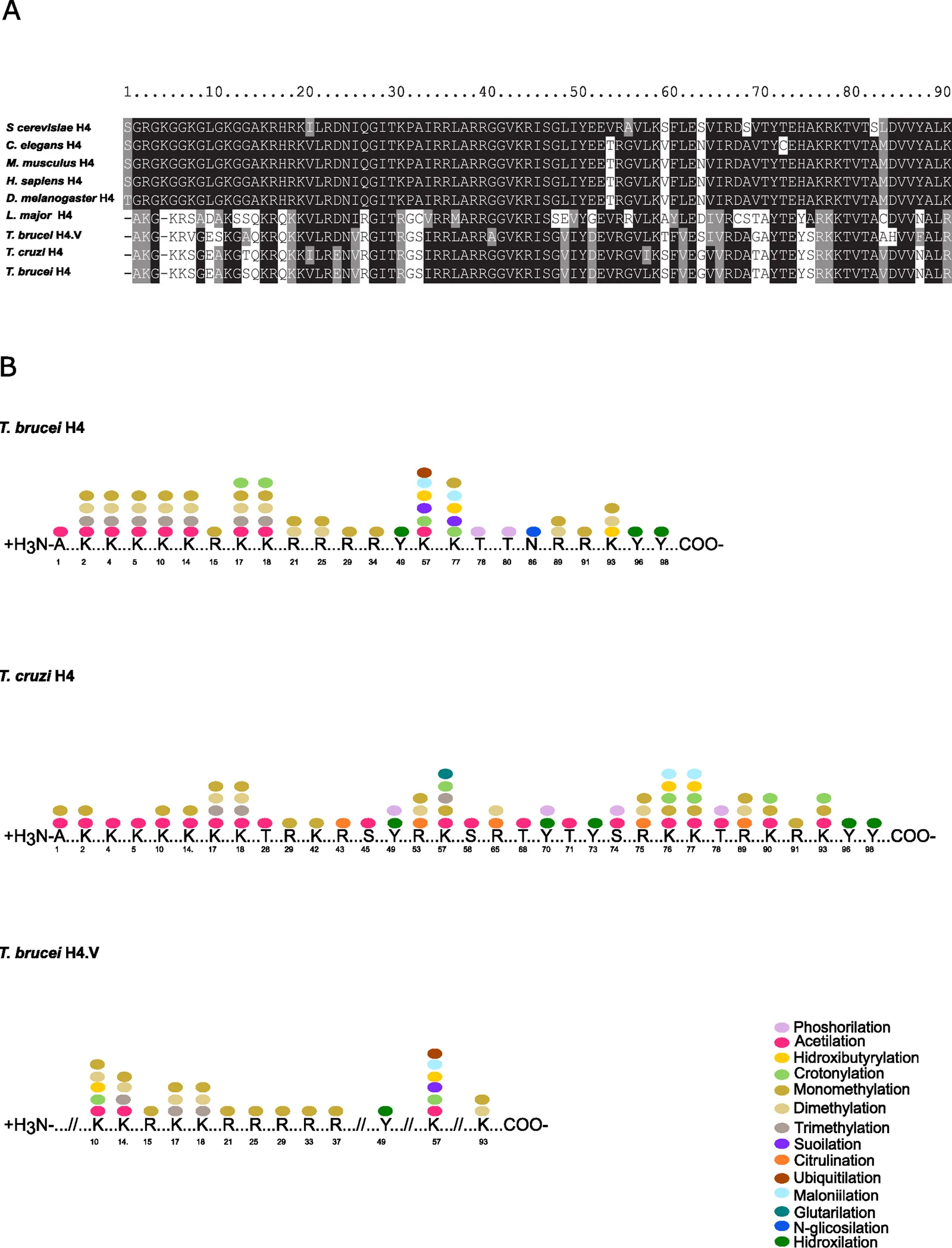
A highly conserved H4 and the unique emergence of H4.V in T. brucei. (**A**) Alignments and shadings were performed with ClustalW and Boxshade 3.3, respectively. The following sequences were used: *L. major* Friedlin: H4 (LmjF.06.0010); *T. cruzi* Dm28c H4 (C4B3_128g301c); *T. brucei* brucei TREU927: H4 (Tb927.5.4260) and H4.V (Tb927.2.2670); *S. cerevisiae:* H4 (NP_009563.1); *Drosophila* H4 (NP_724344.1); *C. elegans* H4 (NP_492641.1); H. *sapiens* H4 (CCDS30847.1); *M. musculus* H4 (CCDS26291.1). Only the first 90 aminoacidic residues of the sequences are shown. (**B**). Schematic representation of the PTMs detected in *T. cruzi* H4, and *T. brucei* H4 and H4.V. The distance between residues is not represented on a real scale. Different colors indicate different PTMs as detailed in the chart.

Despite being a highly conserved histone, the N-terminal tail shows some variations [3] (Figure 5A), but some lysine residues within it are well conserved. Such is the case for lysine residues in positions 2, 4, 10, and 14 in TriTryps; the equivalent residues in model eukaryotes are in positions 5, 8, 12, and 16. These conserved residues are targets for acetylation.

More than 20 or 30 modified amino acid residues were detected in TcH4 and TbH4, respectively. These PTMs are dispersed along the N-terminal tail, the C-terminal tail, and within the globular domain [22,25]. It is worth noticing that at the N-terminal tail, acetylations and methylations prevail in both trypanosomes (Figure 5B). TbH4 is either less extensively modified than TcH4, or some modifications are still elusive.

Remarkably, only *T. brucei* possesses histone variant H4.V in which 12 modified residues have been detected so far, but their roles have yet to be explored [25].

One peculiarity of this parasite is that throughout the cell cycle, it does not go through intracellular stages. This physiological uniqueness and the associated need to switch the expression of its variant surface glycoproteins (VSG) might have pushed throughout evolution for the emergence of a specialized H4.V. As a matter of fact, H4.V deletion, together with H3.V, leads to changes in chromatin accessibility and to an unusual expression of VSG isoforms [31]. Additionally, H4.V is a major signal for transcription termination, as shown by a panel of null mutants [55].

## Unveiling the epigenetic code of highly divergent eukaryotes

### Genomic distribution of histone variants, histone PTMs, and their potential roles

**Figure 6:**
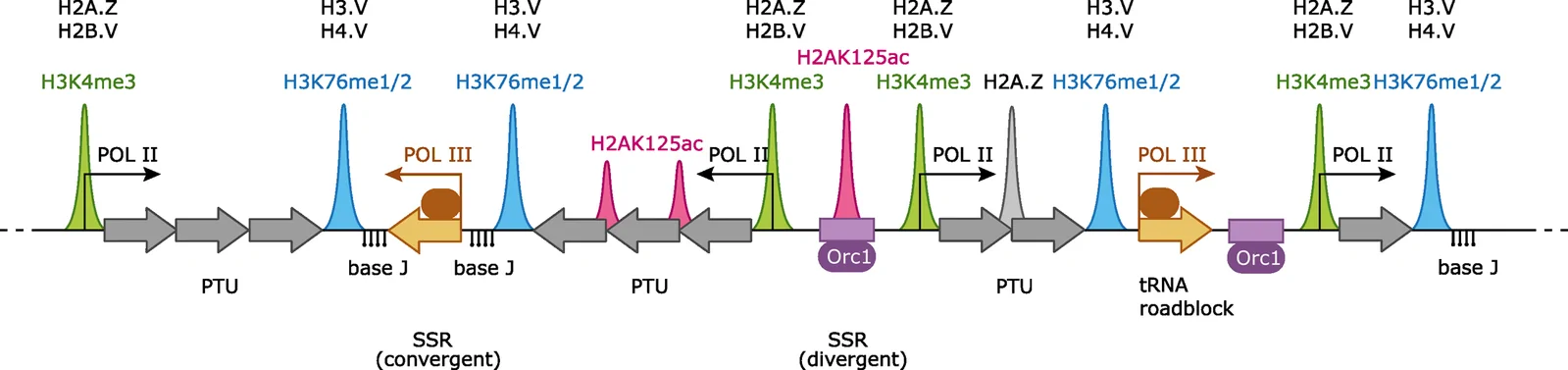
Genomic distribution of the most relevant epigenetic marks in *T. brucei.* Figure adapted from Mareé et al.., B.B.A., 2014, highlighting the best-characterized epigenetic marks described so far in *T. brucei*. Transcription mediated by Pol II starts from disperse promotors normally enriched in H2A.Z/H2B dimer, H3K4me3, and H2AK125ac. H2A.Z was also detected at some internal TSS. TTS are characterized by the presence of base J, H3.V and H4.V. POL II: polymerase II, Orc1: Origin Recognition Complex subunit 1 (TbORC1/CDC6 components), tRNA roadblock: hypothetical tRNA gene representing a roadblock to pol II transcription within a PTU, SSR: strand switch region.

Numerous PTMs have been detected in every canonical and variant histone among TriTryps, but their genomic distributions or potential roles have been poorly explored. The most relevant ones and best characterized are summarized in Figure 6 and discussed below.

In model eukaryotes, H2A.Z localizes around transcription start sites (TSSs), in the +1 and −1 nucleosomes, and it is involved in transcriptional control, DNA repair, and centromeric heterochromatin regulation [56]. In *T. brucei,* H2A.Z was found for the first time to dimerize with a noncanonical histone H2B.V and to be enriched at repetitive DNA [37]. With the emergence of genome-wide technologies, the precision of mapping was refined and the H2A.Z/H2B.V dimer was found at the beginning of the polycistronic transcription units that work as TSSs in TriTryps [57]. Combination with site-specific analysis revealed that the correct deposition of H2A.Z is mediated by GT-rich sequences located in promotor regions [58].

Consistent with a role in facilitating transcription initiation, H2A.Z/H2B.V-containing nucleosomes colocalize with H4K10Ac and are less stable than nucleosomes formed by their canonical or unmodified histone counterparts [6]. In model organisms, a similar less stable nucleosome at the TSS requires H2A.Z and H3.3 [59], while in *T. brucei,* H3.V seems to be involved in a different role, discussed below. As expected for such an important role, genes encoding TbH2A.Z and TbH2B.V are essential for cell viability [37]. In the case of TbH2B.V, the essential part resides in the extended sequence that makes the C-terminal tail longer when compared with canonical H2B (Figure 3A) [42]. There are no data for *L. major,* but in *L. tarentolae,* H2A.Z and H2B.V are also essential genes and the H2A.Z/H2B.V dimer displays a similar genomic distribution [29,60].

In *T. cruzi,* TcH2A.Z was not studied yet, but TcH2B.V is enriched at some tDNA loci, between the so-called conserved and disrupted genome compartments and at putative TSSs, suggesting a conserved genomic distribution among TriTryps [28]. Besides, TcH2B.V was differentially detected during the life cycle, being enriched in tissue-derived trypomastigotes (TCTs). This observation suggested that TcH2B.V may contribute to the differences in chromatin structure and global transcription rates observed among the parasite life forms [23,61]. Not only the presence of TcH2B.V has a life cycle stage-specific association but some PTMs seem to be finely tuned such as TcH2B.VK65me1, which is detected in a specific time frame [23]. However, whether TcH2B.V itself or its PTMs have a role in metacyclogenesis requires further investigation. Controversially, TCTs have higher levels of TcH2B.V protein than epimastigotes [21,61], but TcH2B.V is poorly detected in TCT compared with epimastigotes when analyzed by ChIP-seq [28]. Although the authors have shown some evidence for a weaker interaction of TcH2B.V with chromatin in trypomastigotes compared with epimastigotes, more extensive research will be required for a better understanding of this matter.

An interesting hot spot for PTMs is the C-terminal tail of TbH2A, which seems to be important for acetylation-mediated signaling. TbH2AK125ac peaks are enriched at TSSs, at regions where Pol II transcription terminates, and along the PTU, while TbH2AK115 is particularly detected at the spliced-leader (SL) array [25].

Another histone mark conserved among eucaryotes, which is normally associated with active chromatin, is H3K4me3 [62]. In *T. cruzi*, this mark is present at divergent strand switch regions associated with the TSS [30]. In *T. brucei,* TbH3K4me3 is also located at TSS associated with Pol II-mediated transcriptional events and colocalizes with TbH2A.Z/TbH2B.V and TbH4Kac [6,26,42]. In *L. major*, H3 acetylation is especially enriched at TSSs [52]. In *L. tarentolae,* H3K4me3 is also located at TSSs, colocalizing with H3K16ac and H3K76me3 [29].

As pointed out above, H3K76 differential methylation demands special attention for being involved in cell cycle progression [21,32,33]. In *T. brucei*, these marks are enriched at origins of replication and transcription termination sites (TTS), as detected by ChIP on ChIP [53]. In *T. cruzi*, apart from the above-described role in cell cycle progression, H3K76 differential methylation seems to be closely connected to the parasite life cycle. TcH3K76me1 and TcH3K76me2 are more abundant in replicative forms and TcH3K76me2 shows a tendency to increase during differentiation from epimastigotes to cell-derived trypomastigotes [21,23]. However, the genomic locations of these marks are still unexplored. In *L. tarentolae*, H3K76me3 was detected at TSSs. It is worth mentioning that apart from being differentially methylated, H3K76 is also the target of some other PTMs, such as crotonylation or acetylation and ubiquitylation, whose roles are still elusive.

Among the best-characterized modifications on H4, acetylation stands out and was found to be enriched at TSSs in the three parasites [6,30,63]. In *T. cruzi,* these PTMs were associated with cell cycle progression differentiation and DNA damage response [34]. Additionally, expressing a nonacetylatable version of histone H4 lysines 10 or 14 distorts transcription and replication. The modified histones differ in their capacity to interact with the chaperone Asf1, suggesting that H4K acetylation might be required to establish partner with this protein [64]. Moreover, TcH4K10ac and TcH4K14ac levels are decreased after nutritional stress of epimastigotes and increased during metacyclogenesis [23].

Finally, H3.V and *T. brucei* H4.V constitute specialized histone marks. In *T. cruzi*, the role and genomic distribution of H3.V is still uncharacterized. In *L. tarentoalae,* H3.V is located at TTSs [29]. In *T. brucei,* TbH3.V, together with TbH4.V, is enriched at TTSs [6]. Depletion of H3.V causes antisense transcription in genes neighboring the TTS and leads to a switch in the expression of VSG, changing DNA accessibility [31,65]. Additionally, it was recently shown that H4.V is the major signal for transcription termination [55].

These histone variants, together with H2A.Z and H2B.V, established a chromatin-based transcriptional punctuation [57]. Since most of the regulation in TriTryps occurs post-transcriptionally, this discovery represented a turning point in our knowledge.

Besides the code mediated by histones marks, the presence of tRNA genes nearby acetylated histones at strand switch regions has been proposed to act as a roadblock for Pol II [66]. Adding to the complexity of chromatin punctuation in *T. brucei*, components of the Origin Recognition Complex (ORC1) present a chromatin footprint between replication origins and the boundaries of the PTUs. This genomic distribution suggests a connection between DNA replication and transcription in trypanosomes [67].

### Cross-talk among epigenetic marks and functional implications

Deciphering the histone code of each eukaryote is an ongoing work, but especially in TriTryps, this field is at the onset of our knowledge. The role of most histone marks is unsolved, and, in many cases, it is hard to unveil their roles. Especially, it is considered that they work as part of a co-ordinated network. As introduced in the above section, the simultaneous presence of different histone marks and the co-ordinated interaction with other proteins such as readers, writers, and erasers are frequently required.

A recent study by Mareé and colleagues implemented a proteomic approach that generates longer peptides to identify coexisting histone PTMs and highlights the relevance of TbH2A C-terminal tail hyperacetylation. In parallel, those modifications were detected at specific genomic regions suggesting that differential hyperacetylation of the H2A C-terminal lysine residues could have different epigenetic functions [25]. While H2AK115 peaks were found within and between PTUs and showed stronger signals in the blood form of the parasite, H2AK125 peaks were mainly detected at the SL loci, at divergent strand switch regions and intergenic regions.

A well-studied example, in *S. cerevisiae,* is the ubiquitination of H2B lysine 123. This modification is required for methylation of H3K79 [68,69]. Moreover, it has been proposed that H2B ubiquitination acts as a glue to favor the activity of SET1 and DOT1 to methylate their target residues (H3K4 and H3K79, respectively) on the nucleosome [70]. However, even though TbH2BK129 is a homolog of *S. cerevisiae* H2BK123, this residue is not ubiquitinated in the parasite and its mutation does not affect H3K4 or H3K76 methylation. Furthermore, the heterologous expression of *T. brucei* DOT1a and *T. brucei* DOT1b in yeast revealed that H2BK123 ubiquitination is especially important for DOT1a activity in the heterologous system [71]. This observation points to a mechanism that differs from the one observed in yeast with its endogenous enzymes, where H2B ubiquitination is indispensable for monomethylation either by DOT1 or SET1 [72]. In *T. brucei*, instead, a different cross-regulation takes place. TbH2B.V replaces TbH2B in nucleosomes that are enriched in H3 methylation at residues K4 and K76. Moreover, a deletion of the C-terminal tail of TbH2B.V is lethal. This observation suggests the existence of a distinctive mechanism for H3K4 and 76 methylation in *T. brucei* that demands the presence of H2B.V [42]. It remains unresolved whether TbH2B.V is indispensable for DOT1a activity on H3K76 directly or upon activation of H3K4 methylation, since the coexistence of K4 and K79 methylations was detected, but their potential coregulation is still unexplored. *In vitro* experiments with recombinant enzymes and reconstituted nucleosomes, as well as the characterization of the methyltransferase responsible for H3K4 methylation in this parasite, will be necessary to understand the whole puzzle.

Another cross-talk described in *T. brucei* is the above-mentioned regulation that involves TbH3.V, TbH4.V, and the modified DNA base denominated base J. This impressive discovery showed, for the first time, the implications of epigenetics in transcription regulation in TriTryps [31,65]. More recently, it was pointed out that H4.V is actually the major signal for transcription termination [55].

Acetylations in H4 are among the best-characterized PTMs. In model organisms such as yeast and mammals, acetylations in the N-terminal tail of histone H4 are associated with open chromatin regions around TSS and promote transcription. Moreover, H4 acetylation is important for the correct deposition of histone variant H2A.Z [73]. In *T. brucei*, a very similar regulation takes place. H4K10ac is enriched up to 300-fold at the TSS, alongside the bromodomain factor BDF3 and the heterodimer H2A.Z/H2B.V [6]. The impairment of H4 acetylation affects H2A.Z deposition and causes a shift in RNA pol II transcription initiation sites [74]. *In T. cruzi*, the overexpression of nonacetylatable versions of H4 disrupts transcription and replication [64].

In *T brucei*, 58 PTMs were recently identified at TSSs involving acetylation of H2A.Z and H4 mediated by different histone acetyltransferases. While depletion of histone acetyltransferase 2 (HAT2) leads to a loss of TSS-associated H4 acetylation and to decreased incorporation of H2A.Z into chromatin, depletion of histone acetyltransferase 1 (HAT1) reduces histone H4 acetylation and affects transcription levels [74]. Additionally, it was shown that BDF2 was involved in the interaction with the acetylated N-terminal tail of H2A.Z [75]. H4K10ac colocalized with BDF3, but their interaction was not demonstrated [6]. In *T. cruzi* instead, BDF2 interacts with H4K10ac and H4K14ac [76]. It remains to be explored whether BDF2 could also act as a reader of H2A.Z acetylation as observed in *T. brucei* or if that function is displayed by a different protein.

Moreover, different levels of PTMs at specific time points might be crucial for differentiation along the life cycles in trypanosomes. A quantitative proteomic approach during metacyclogenesis performed in *T. cruzi* revealed that most histone peptides are unmodified. Among the modified ones, methylations prevail over acetylations and monomethylation are the least abundant marks at all time points. Quantification of histone PTMs during metacyclogenesis revealed co-ordinated variations in these histone marks. Among the most striking observations, acetylation of H2A.Z is pronounced at stationary phase, and acetylation of histone H4 follows a continuous increment in their levels along the transition from epimastigotes to metacyclic trypomastigotes. This observation is accompanied by an increment of H3K76me2, suggesting a connection between cell cycle progression and differentiation [23]. These changes in epigenetic marks might be fine-tuning chromatin accessibility. More refined studies for different PTMs will be required to unveil their patterns and potential roles.

## Concluding remarks

The fact that trypanosomes have complex life cycles implies that they need to adapt to different environmental conditions when moving through the different phases. There are many examples in eukaryotes where the environment affects metabolic responses through the epigenetic code, triggering different signaling pathways, and trypanosomes are not an exception [77]. Uncovering the rules of their histone code and highlighting the divergence from their host cells might lead to the discovery of potential new targets for drug development.

Although abundant PTMs have been detected in every canonical and variant histone among TriTryps, their genomic location or potential implications have only been explored for a few of them. More extensive proteomic and genomic analysis will be needed to include *L. m*ajor in a detailed comparison with the trypanosomes. A major stumbling block has been the lack of commercial antibodies for trypanosomatid histones and the complexity of their genomes that harbors a high content of repetitive sequences. The advent of long-read technologies and new-generation genomic editing tools for endogenous gene tagging can help to move the field forward. Upcoming studies mapping the genome-wide distribution of histone PTMs and histone variants will contribute to put the pieces of this puzzle together.

## Abbreviations

ORC1: Origin Recognition Complex
PTMs: post-translational modifications
PTUs: polycistronic transcriptional units
SL: spliced-leader
TSSs: transcription start sites
TTS: transcription termination sites
VSG: variant surface glycoproteins
